# Development of a New Compression Garment Less Affected by Postural Changes and Movement for Lower-Extremity Edema

**DOI:** 10.7759/cureus.81316

**Published:** 2025-03-27

**Authors:** Yukako Ishida, Yukie Takahashi, Kayo Asahi, Shinji Tsukamoto, Akira Kido

**Affiliations:** 1 Rehabilitation Medicine, Nara Medical University, Kashihara, JPN; 2 Orthopedic Surgery, Nara Medical University, Kashihara, JPN

**Keywords:** compression garment, compression therapy, lymphedema, novel shape, venous edema

## Abstract

Background

The principle of edema treatment is conservative, and compression therapy is the mainstay of conservative treatment. In this study, we developed a compression garment with a novel and effective shape and examined its pressure maintenance.

Methods

We focused on the shape of the gap between the pullers, which affects pressure stability. First, we designed models with a smaller gap area by increasing the number of pullers and then selected an optimal number for the new product. Next, we investigated the effects of posture and movement on the interface pressure using the new product in seven healthy participants and statistically analyzed the results.

Results

Based on comparisons between models with reduced gap areas, we determined the number of pullers to be four and developed a new product. Compared with current products, the new product maintained a significantly higher interface pressure and was less affected by posture and movement.

Conclusions

Shape, especially the number of pullers and shape of the gaps, is an important factor determining the therapeutic effect of compression garments for edema treatment, which can maintain a higher interface pressure and reduce fluctuations due to posture and movement. The newly developed compression garment with a novel shape is expected to produce a high and stable therapeutic effect.

## Introduction

Edema is the accumulation of excess fluid in the interstitial spaces of tissues and is common in daily life [1]. Edema can be classified as systemic or localized. Systemic edema is caused by an increase in extracellular fluid due to cardiac or renal failure, whereas local edema is caused by vein and lymphatic vessel disorders. Local edema includes lymphedema, in which abnormal protein-rich fluid accumulates due to dysfunction of the lymphatic system [1], and venous edema, in which blood flow in the superficial and deep veins is obstructed by chronic venous insufficiency, resulting in venous hypertension [2]. More than 90% of patients are women, and lymphedema often develops after surgery for gynecological diseases, such as uterine, ovarian, and breast cancers [1]. Both types of edema are progressive, and once they develop, they are often difficult to treat. Complications, such as cellulitis, hyperpigmentation, and ulceration, can occur, leading to refractory healing and a decline in activities of daily living and quality of life.

Conservative treatment is the mainstay of edema treatment, although drug therapy and surgery are also used [1]. The mainstay of conservative treatment is compression therapy, in which elastic bandages, stockings, and other elastic garments are used. Compression therapy is expected to reduce edema by applying moderate pressure to the affected limb using bandages and elastic garments. For venous edema, the application of a compression pressure of at least 15 mmHg at the ankle-joint level is recommended [3,4,5]. For lymphedema, the International Society for Lymphology recommends a compression pressure of 20-60 mmHg as the highest pressure within the range acceptable to the patient [1]. The International Society for Lymphology also recommends complex decongestive physiotherapy, which consists of compression therapy using elastic garments, exercise therapy, manual lymphatic drainage, and skin care, as an effective conservative treatment for lymphedema [6,7]. Several reports have been published on the number of layers and elasticity of bandages used in compression therapy [8,9]. The adjustable feature has attracted attention when comparing them, and the usefulness of Vercro®-type supporters, which have more freedom of shape than bandages, has been established [10-13]. This was also the starting point for our study.　

At our university hospital, a lymphedema outpatient clinic was established in September 2016, led by two nurses certified as specialized lymphatic drainage therapists. The main target is lymphedema after gynecological cancer surgery. However, the clinic also treats patients with venous edema. The mainstay of treatment is compression therapy with elastic garments and manual lymphatic drainage. The nurses instruct patients on applying the garment at the appropriate pressure, using the measured pressure and physical findings as indicators.

In 2023, we began trialing the use of a ready-made “calf supporter” (Takagi, Nara, Japan) as a simple and adjustable compression garment for lower leg. It is a fully sewn Velcro®-type supporter. The side that comes in contact with the skin is covered with a satin net to improve the sensation against the skin. There are two sizes, medium (M) and large (L); the garment of size M has two pullers, whereas that of size L has three. The pullers are wrapped around the patient's limbs to apply pressure. However, when we tested the product on patients attending the lymphedema outpatient clinic, we found the following: (1) it was difficult to choose the right size, and (2) there were gaps between the pullers, making it difficult to apply the correct amount of pressure and causing the product to slip.

To achieve a better therapeutic effect, it is necessary to maintain a stable pressure. Therefore, we considered the following two improvements: (1) modifying the garment to a single size by extending the length and width (with the intention of cutting and adjusting the size as required) and (2) increasing the number of pullers and reducing the area of gaps. The purpose of this study is to develop a new garment with a more ideal gap shape and to evaluate whether it provides a higher pressurization effect compared to current products.

## Materials and methods

Compression garment

The current product, “calf supporter”(Takagi Co., Nara, Japan), is a Velcro®-type supporter that can be adjusted for pressure by wrapping the puller around the lower leg. The outer fabric is composed of 87% nylon and 13% polyurethane, whereas the inner fabric is composed of 95% nylon and 5% polyurethane. The surface that touches the skin is covered with satin net for a good sensation against the skin, and there are no seams touching the skin because the product is completely sewn without seams. The product was available in two sizes: M and L. The M size is 29.0 cm long and 31.7-44.5 cm wide, with two pullers, and is suitable for patients with a lower-leg circumference of 24-38 cm. The L size is 35.4 cm long and 31.6-47.3 cm wide, with three pullers, and is suitable for patients with a lower-leg circumference of 35-46 cm. During the trial period at our hospital from 2023, as mentioned above, issues with this product were noticed, such as difficulty in choosing the size and gap formation between the pullers, making it difficult to apply moderate pressure and causing the product to slip. Therefore, we developed a new product with a more stable therapeutic effect. This study began on April 1, 2023, and ended on February 28, 2025.

Shape development strategy

**Figure 1:**
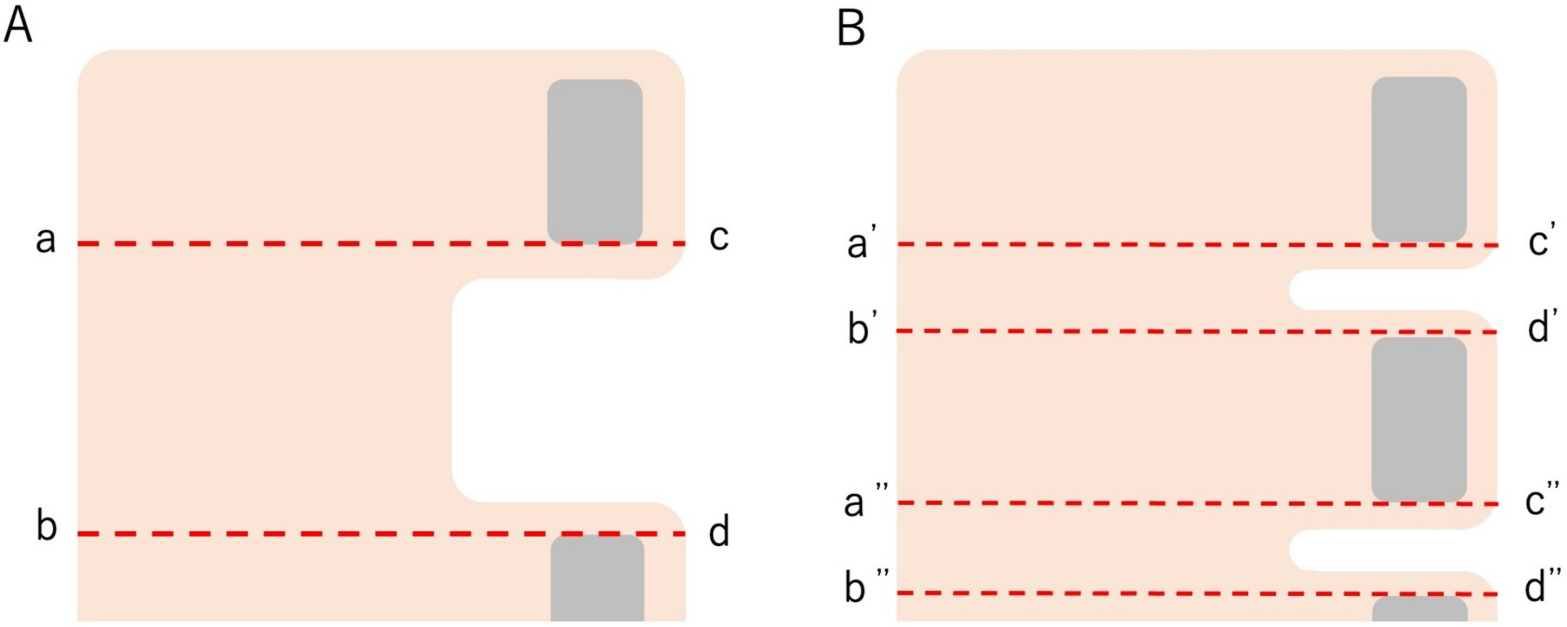
A schematic of a garment with multiple pullers. If we reduce the gap area, we can make the pressure more stable. B shows the state where the number of pullers increased from A.

Figure 1 is a schematic of a garment with multiple pullers. Conventional garments have two or three pullers (M and L, respectively). The area enclosed by points abcd is a gap where there is no horizontal Velcro®, and we hypothesized that if we could reduce the area of this gap, we could reduce the variation in stress distribution and make the pressure more stable (B shows the condition where the number of pullers has been increased from A). There are two ways to reduce this gap: either by narrowing the gap without increasing the number of pullers or by increasing the number of pullers and dividing the gap into smaller sections. Considering that this garment would gradually squeeze lymph fluid or blood from the distal extremities when worn, we decided to increase the number of pullers. First, we made a model with four or five pullers to reduce the gap, and then we planned to measure the pressure using these models. The long axis length is equal to size L. If it was predicted that it would be better to increase the number of pullers further, we planned to make a model with even more pullers (model selection).

Participants

Seven healthy women participated in this study. Their ages ranged from 23 to 44 (average age 29.71 ± 7.32) years. The purpose and content of the study were explained to each participant, and cooperation was requested after they understood the details. Participation in the study was a matter of free will. Written informed consent was obtained from all individual participants enrolled in the study. This study was approved by the Ethics Committee of Nara Medical University (approval no. 3976).

Measurements

Height, weight, body mass index (BMI), grip strength, lower-leg circumference, extracellular water-to-total body water ratio (ECW/TBW), and pressure when wearing the supporter were measured. The lower-leg circumference was measured as the maximum circumference of the lower leg. InBody® S10 (InBody Japan, Tokyo, Japan) was used to measure the ECW/TBW. PicoPress® (Microlab Elettronica, Ponte San Nicolò, Italy) was used to measure the interface pressure between the body and garment.

First, in the model selection stage, following the shape development strategy described above, there were one subject. The measurement sites were the maximum circumference of the lower leg and the transition area between the Achilles tendon and gastrocnemius muscle. Five measurements were taken in the supine and standing positions, and the average values were recorded.

Next, measurements were taken under the following conditions for the new product, whose shape was selected based on the model comparison. The sensor was placed in the transition area between the Achilles tendon and gastrocnemius muscle. Measurements were taken in the following positions: supine, standing, squatting (knee joint flexion: 30°), and standing with the ankle joint dorsiflexed, toes flexed, and on the toes. For supine and standing, the posture was maintained until the pressure stabilized, and the most stable 10-second value was averaged. For squat, ankle dorsiflexion, toe flexion, and tiptoe, each exercise was performed five times and the values averaged.

Statistical analysis

The Mann-Whitney U test was used to compare the measurement values between postures. The Wilcoxon signed-rank sum test was used to compare the current and new products. Statistical significance was set at P < 0.05. Data were analyzed using JMP Pro version 17.2.0 (SAS Institute Inc., Cary, NC, USA).

## Results

Participant characteristics

Table 1 presents the characteristics of the participants. The average height, weight, and BMI of the healthy volunteers were 160.21 ± 3.66 cm, 49.43 ± 3.81 kg, and 19.25 ± 1.21 kg/m^2^, respectively. The average lower-leg circumference on the side which the garment was worn (right) was 34.00 ± 1.20 cm. The average ECW/TBW was 0.382 ± 0.004, which was within the standard range. The average grip strength was 30.77 ± 2.81 kg on the right and 27.37 ± 2.83 kg on the left.

**Table 1:** Characteristics of the participants. BMI: body mass index, ECW/TBW: extracellular water to total body water ratio

No.	Age(years)	Height（m）	Weght（kg）	BMI（kg/m^2^）	Calf circumference（cm）	ECW/TBW
1	23	1.57	50	20.28	35.0	0.378
2	31	1.6	53	20.7	35.5	0.388
3	44	1.575	47	18.95	33.0	0.389
4	36	1.63	45	16.94	32.5	0.378
5	23	1.55	45	18.73	32.5	0.376
6	24	1.63	50	18.82	34.5	0.381
7	27	1.66	56	20.32	35.0	0.381

Comparison of models with different numbers of pullers

**Figure 2:**
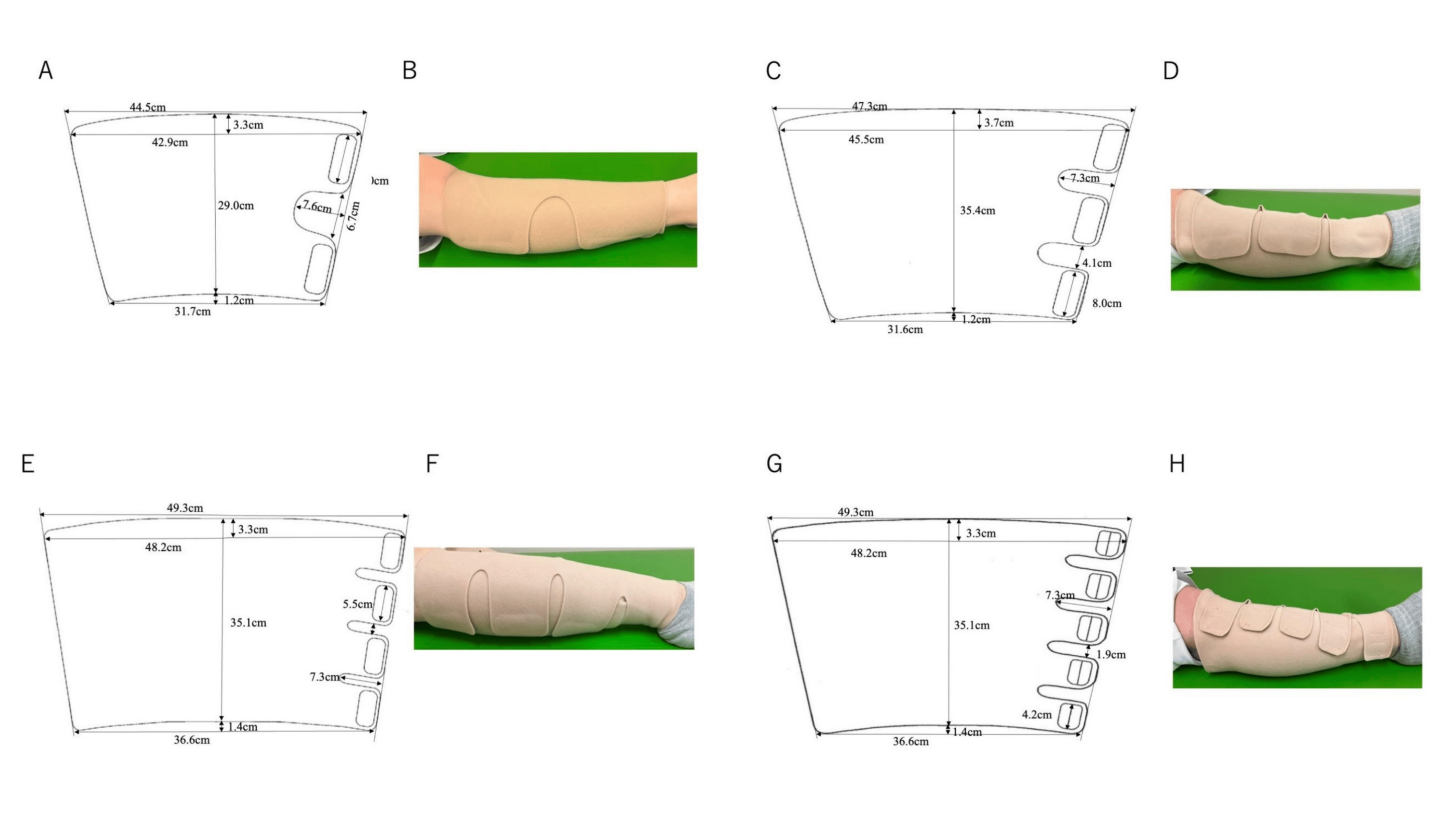
Geometry and appearance of garments with two pullers (A and B, current product, size M), three pullers (C and D, current product, size L), four pullers (E and F) and five pullers (G and H). Two pullers and three pullers are currently used in clinical practice. Four pullers and five pullers are models designed in this study to explore the optimal number of pullers.

**Figure 3:**
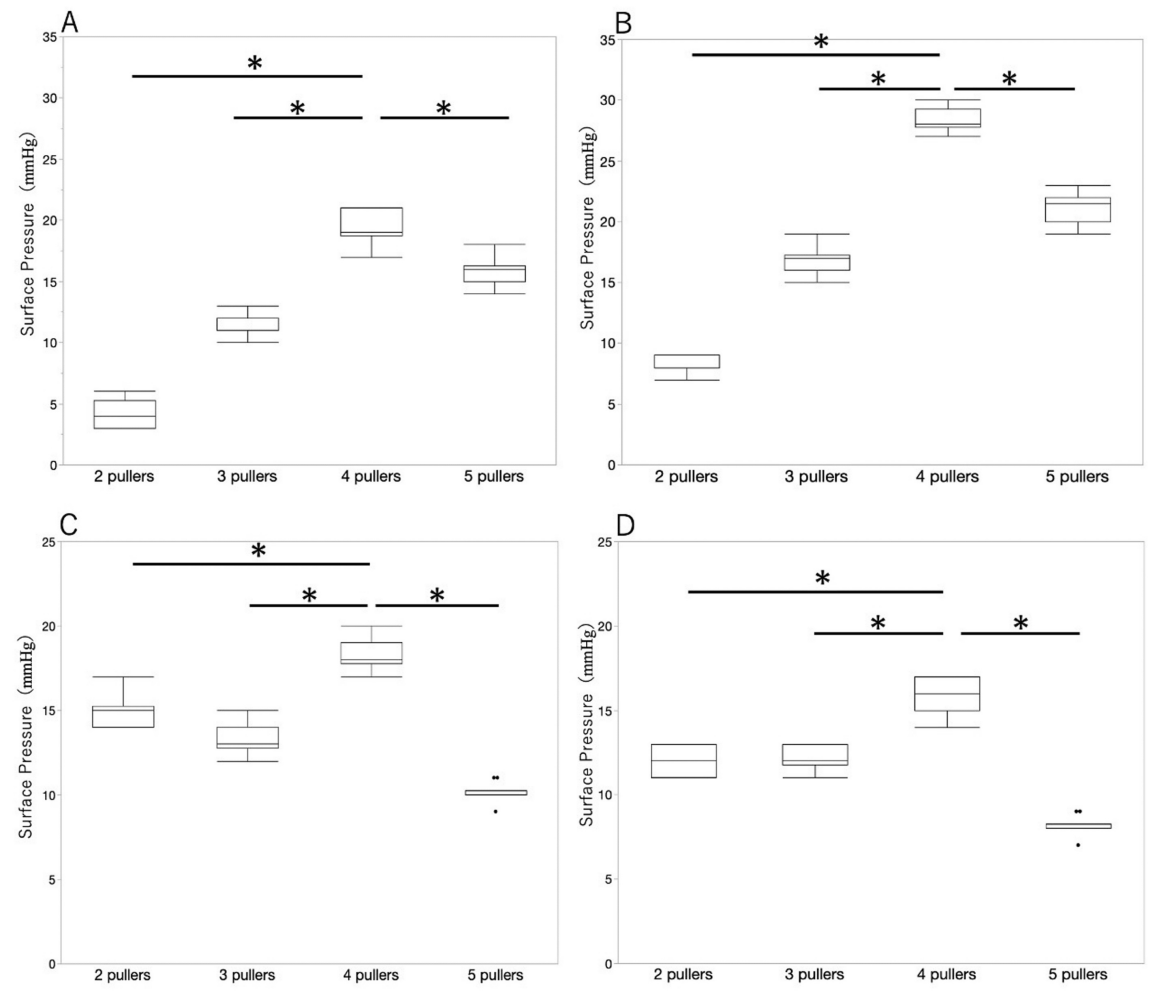
Comparison of surface pressure in supine and standing positions for the garment with two pullers (current product, size L), three pullers (current product, size M), four pullers, and five pullers. These graphs compare the model designed in this study to current products. They show the pressure at the transition area between the Achilles tendon and the gastrocnemius muscle in supine (A) and standing (B) and the pressure at the part of the maximum circumference of the lower leg in supine (C) and standing (D). The pressure was significantly highest with four pullers and decreased with five pullers. Based on these results, we designed a new product with four pullers. The Wilcoxon signed-rank sum test was used to compare the measurement values of the products with two pullers, three pullers, four pullers, and five pullers. *p < 0.05

Figure 2 shows the geometry of the conventional product with two or three pullers and the model with four or five pullers. These models were designed according to the strategy of reducing the gap area. Figure 3 shows the results of measurements on a healthy person (Table 1, No. 7) for these models. In both the supine and standing positions, the pressure was highest with four pullers and decreased with five. Based on these results, we created a new product with four pullers. 

Interface pressure of the current and new products

**Figure 4:**
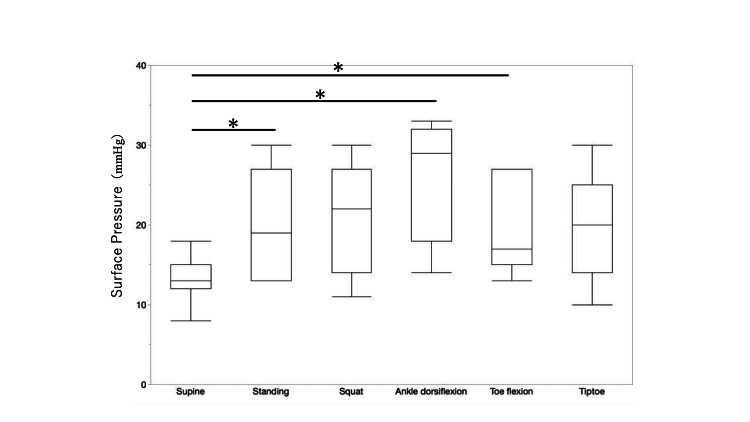
Surface pressure in the supine position, standing position, and during movement when wearing the current product. Compared with the pressure in the supine position, the pressure in the standing position, standing with the ankle joint dorsiflexed, and standing with the toes flexed were significantly higher. The Mann-Whitney U test was used to compare the measurement values between postures. *p < 0.05

Figure 4 shows the interface pressure of the current product. The average pressures in various positions were as follows: supine position, 13.4 ± 3.26 mmHg; standing position, 20.4 ± 6.65 mmHg; during squatting, 20.86 ± 6.53 mmHg; standing with the ankle joint dorsiflexed, 25.14 ± 7.28 mmHg; standing with the toes flexed, 19.43 ± 5.45 mmHg; and standing on the toes, 19.86 ± 6.31 mmHg. The average pressure was lowest in the supine position and highest during standing with the ankle joint dorsiflexed. Compared with the pressure in the supine position, that in the standing position and during standing with the ankle joint dorsiflexed and standing with the toes flexed were significantly higher.

**Figure 5:**
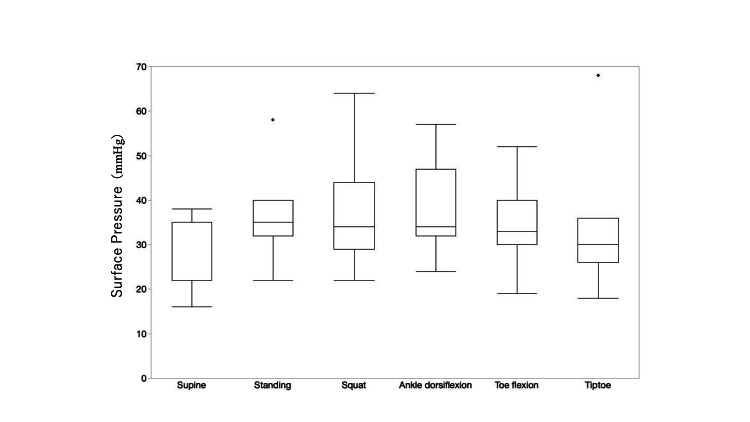
Surface pressure in the supine position, standing position, and during movement when wearing the new product. There was no significant difference when compared with the average pressure in the supine position. The Mann-Whitney U test was used to compare the measurement values between postures.

Figure 5 shows the interface pressure of the new product (with four pullers). The average pressures in the various positions were as follows: supine position, 24.6 ± 7.31 mmHg; standing position, 32.2 ± 5.64 mmHg; during squatting, 37.43 ± 12.70 mmHg; standing with the ankle joint dorsiflexed, 38.71 ± 10.50 mmHg; standing with the toes flexed, 35.0 ± 9.56 mmHg; and standing on the toes, 34.29 ± 14.86 mmHg. As with the current product, the average pressure in the supine position was the lowest and that during standing with the ankle joint dorsiflexed was the highest; however, there was no significant difference when compared with the average pressure in the supine position.

**Figure 6:**
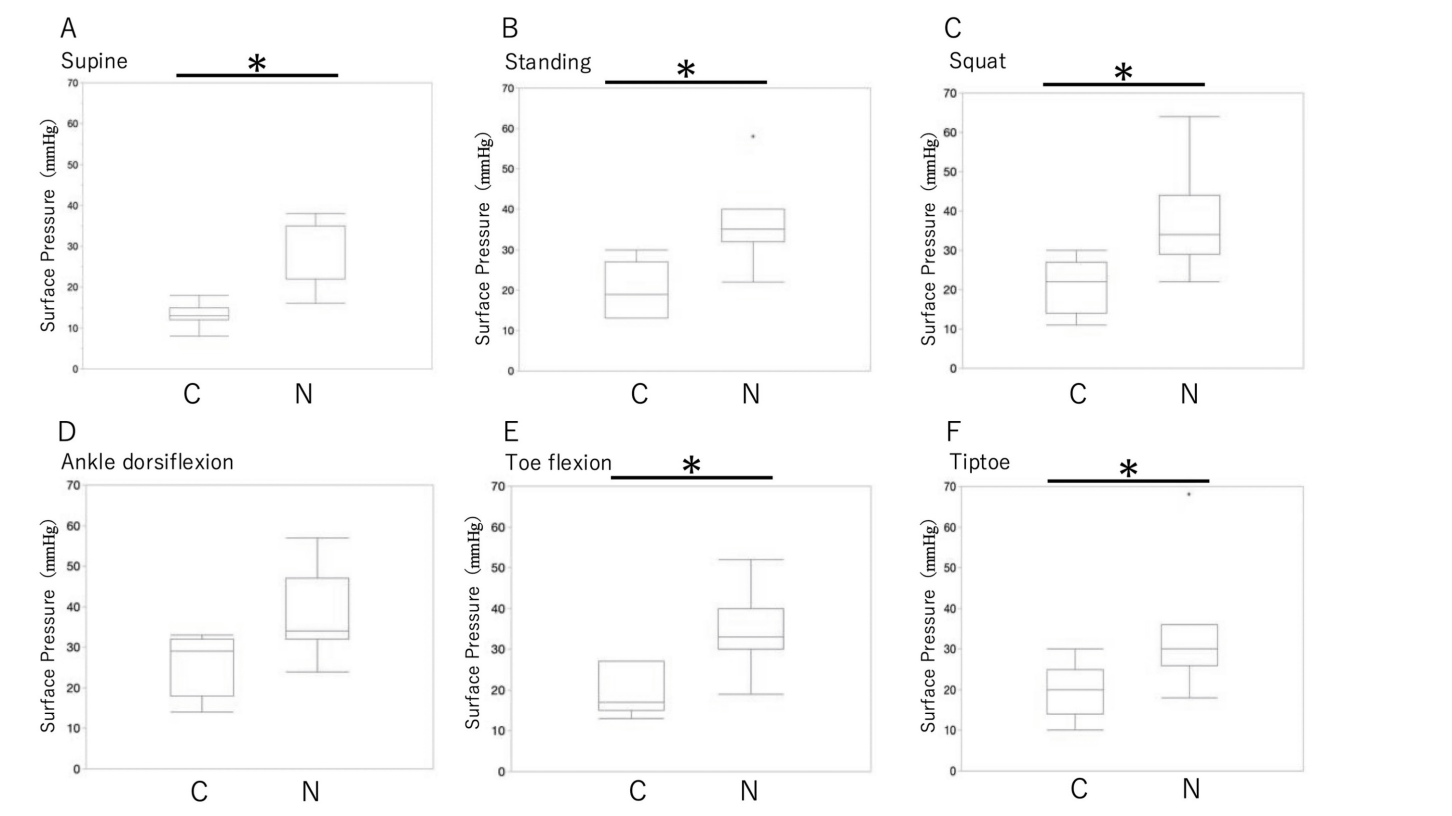
Comparison of surface pressure between the current (C) and new (N) products. The pressure of the new product was significantly higher than that of the current product in supine, standing, squatting, standing with toes flexed, and standing on the toes. The Wilcoxon signed-rank sum test was used to compare the current and new products in supine (A), standing (B), squatting (C), standing with ankle dorsiflexed (D), standing with toes flexed (E), and standing on toes (F). *p < 0.05

Figure 6 compares the pressures of the current and new products. The pressure of the new product was significantly higher than that of the current product in the following positions: supine, standing, squatting, standing with toes flexed, and standing on the toes.

## Discussion

The main treatment for lower-limb edema is conservative treatment using compression therapy. Older patients find it difficult to apply compression using elastic bandages by themselves, and it is sometimes difficult to achieve the desired effect. For these patients, adjustable compression garments have been reported to be effective [14,15]. However, reports also suggest that it is difficult for older people aged 65 years and over to use compression garments at the appropriate pressure owing to factors such as reduced grip strength and cognitive impairment [16]. In addition, it may be necessary to try multiple sizes of a product depending on age and disease severity [17], which can also cause financial burden. Our new product was developed in one size based on the idea of “one size fits all.” It was designed with the intention of making it possible to adjust the size, by cutting the garment, to suit each individual patient. In addition, the design using four pullers made it possible to reduce the gaps, making the product less likely to slip, compared with conventional garments, and it was thought that it would be possible to maintain a high compressive pressure.

In compression therapy, the pressure may differ greatly between rest and exercise and between the supine and standing positions; therefore, it is necessary to consider the effects of posture and movement for each condition and product [18]. One group reported that the pressure was twice as high during dorsiflexion of the ankle joint in the standing position than during plantar flexion; however, this is also affected by the elastic properties of the compression garment [3]. Regarding elastic properties, studies comparing products with different extensibilities have reported that posture can affect pressure in all cases, but short-stretch products are preferable [19-21].

In this study, the pressures during standing, standing with the ankle joint dorsiflexed, and standing with the toes flexed were significantly greater than that in the supine position for the current product. However, there were no significant differences between the pressures in these same postures for the new product. In addition, when comparing the current and new products during the same positions, a significant difference was observed in all cases, except during standing with the ankle joint dorsiflexed. The new product showed an appropriate pressure of 24 mmHg or more from the resting state, and there was no difference in pressure between the various positions. By contrast, the current product did not reach the 15-mmHg pressure at rest needed for venous edema; however, the pressure increased when assuming various positions. In other words, standing, standing with the ankle joint dorsiflexed, and standing with the toes flexed were required to maintain the appropriate compression pressure with the current product, whereas the new product could provide the appropriate compression pressure even at rest.

This research is an industry-academia collaboration between our university and a manufacturer with the aim of improving compression garments that are already in use for therapeutic purposes. We developed a garment with a novel shape by reflecting on the opinions of patients at a lymphedema outpatient clinic who used the current product. As there are various possible variations for improving the shape, further investigation is required to determine the optimal number of pullers and sizes. However, we designed the product prioritizing the feasibility of the manufacturing process and verified the performance of the new product. In addition, one of the themes of this garment development was to adjust the size by cutting; however, the pressure during cutting was not measured; therefore, verification will be necessary in the future.

This study has some limitations. First, the sample size was small. Second, the participants were healthy volunteers; the product has not been verified for effectiveness in patients with lower-limb edema. Third, because the measurement time was short, it is unclear whether the product is effective over a long period of time. However, we hope to improve the effectiveness of the current product. A strength of the current study is that, as this was industry-academia collaborative research, the new product developed is scheduled for clinical use in the near future.

## Conclusions

In this study, we first considered the problems of existing products with two or three pullers. Then, we manufactured and measured products with four or five pullers, using the strategy of reducing the area of the part not sandwiched by the pullers. The four pullers product was the most effective and was tested on healthy subjects in each posture to confirm its good effect. Measurements taken from seven healthy participants revealed that the new compression garment was less affected by posture changes and movement and could maintain a higher pressure than the current product. The shape of the compression garment is an essential factor, and this new product is expected to provide sufficient resting pressure and sound therapeutic effects for lower-extremity edema.
